# A chromium tricarbonyl complex featuring the 4,6-bis(diphenylphosphinomethyl)dibenzothiophene (PSP^Ph^) ligand

**DOI:** 10.1007/s00706-016-1707-9

**Published:** 2016-03-14

**Authors:** Matthias Mastalir, Clara Schweinzer, Matthias Weil, Ernst Pittenauer, Günter Allmaier, Karl Kirchner

**Affiliations:** Institute of Applied Synthetic Chemistry, Vienna University of Technology, Getreidemarkt 9/163-OC, 1060 Vienna, Austria; Institute of Chemical Technologies and Analytics, Vienna University of Technology, Getreidemarkt 9, 1060 Vienna, Austria

**Keywords:** Pincer ligands, Dibenzothiophene, Chromium complexes, Carbonyl ligands

## Abstract

**Abstract:**

The new PSP pincer ligand 4,6-bis(diphenylphosphinomethyl)dibenzothiophene (PSP^Ph^) was prepared in 89 % yield. With this ligand, a solvothermal synthesis of a Cr complex of the type [Cr(κ^3^*P,S,P*-PSP)(CO)_3_] is described. The X-ray structure of this compound is presented. We demonstrate that the solvothermal synthesis technique provides a powerful, simple, and practical synthetic method resulting in a high isolated yield in a short reaction time.

**Graphical abstract:**

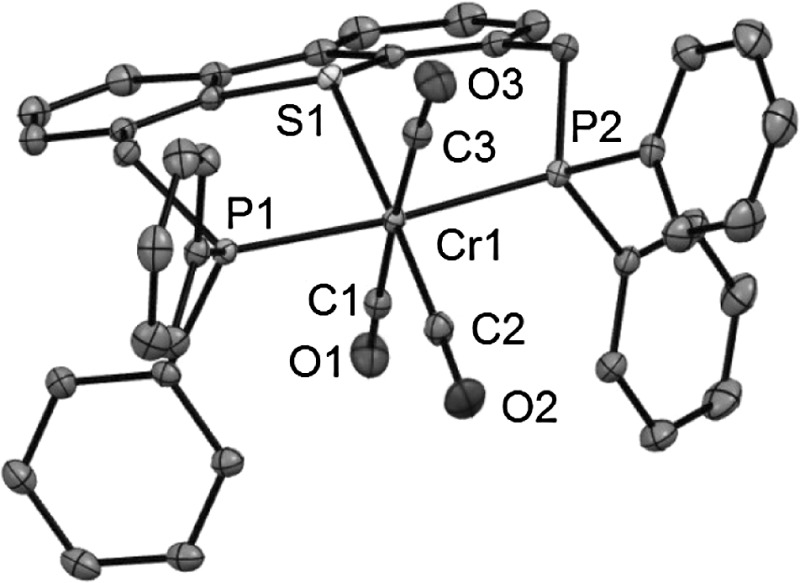

**Electronic supplementary material:**

The online version of this article (doi:10.1007/s00706-016-1707-9) contains supplementary material, which is available to authorized users.

## Introduction

Among the many ligand systems that can be found in the chemical literature pincer ligands play an important role and their complexes have attracted tremendous interest due to their high stability, activity, and variability [1–5]. Pincer ligands are often planar scaffolds consisting of an anionic or neutral central aromatic backbone tethered to two, mostly bulky, two-electron donor groups by different spacers where steric, electronic, and stereochemical parameters can be manipulated by modifications of the substituents at the donor sites and/or the spacers. Phosphine-based PCP and PNP type ligands having central C and N donors have received the most attention. Accordingly, many applications of mostly precious second and third row transition metal pincer complexes in the fields of catalysis, molecular recognition, and supramolecular chemistry were discovered turning this area into an intensively investigated subject in organometallic chemistry.

In the present contribution we report on the synthesis and characterization of a new PSP pincer ligand based on dibenzothiophene, and describe a simple solvothermal synthesis of a chromium tricarbonyl complex bearing this ligand. It has to be noted that tridentate bis-phosphine ligands with a central S donor (PSP ligands) are extremely rare [6].

## Results and discussion

The new pincer ligand 4,6-bis(diphenylphosphinomethyl)dibenzothiophene (PSP^Ph^) (**3**) was prepared from 4,6-bis(hydroxymethyl)dibenzothiophene (**1**) which was converted to 4,6-bis(bromomethyl)dibenzothiophene (**2**) upon bromination with PBr_3_. It has to be noted that this intermediate was generated previously by bromination of 4,6-dimethyldibenzo[*b*,*d*]thiophene with *N*-bromosuccinimide [7]. Diphenylphosphine was then reacted with *n*-BuLi and subsequently treated with **2** to afford, after workup, the PSP ligand 4,6-bis(diphenylphosphinomethyl)dibenzothiophene (PSP^Ph^) (**3**) in 91 % isolated yield (Scheme 1). This ligand was not described before, only the related phosphine oxide [8] as well as a *P*-chiral bis(phosphine-boranes) derivative bearing *t*Bu and Ph substituents [9] were reported recently. Compound **3** was fully characterized by ^1^H, ^13^C{^1^H}, and ^31^P{^1^H} NMR spectroscopy, HRMS, and elemental analysis.

**Figure:**
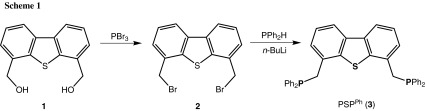

In order to obtain group 6 tricarbonyl complexes with the new PSP pincer ligand **3**, a simple and fast solvothermal approach with no need for a microwave equipment was chosen, which we developed recently for the synthesis of zero valent Cr, Mo, and W complexes [M(PNP)(CO)_3_] with PNP pincer ligands based on the 2,6-diaminopyridine scaffold [10]. Accordingly, a suspension of hexacarbonyl complexes [M(CO)_6_] and **3** in CH_3_CN were placed in a sealed microwave glass tube and stirred for 6 h at 140 °C. From these three precursors only [Cr(CO)_6_] underwent the desired reaction, while in the case of molybdenum and tungsten surprisingly only intractable materials were recovered. After workup, the analytically pure complex [Cr(κ^3^*P,S,P*-PSP^Ph^)(CO)_3_] (**4**) was obtained in 89 % isolated yield (Scheme 2). This complex is air sensitive both in solution and in the solid state.

**Figure:**
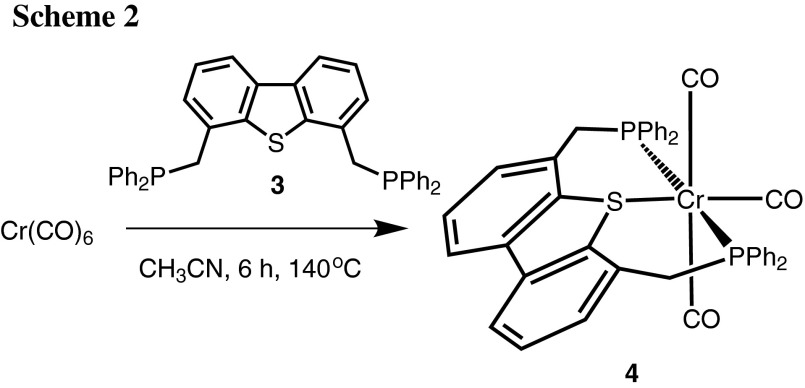

**Fig. 1:**
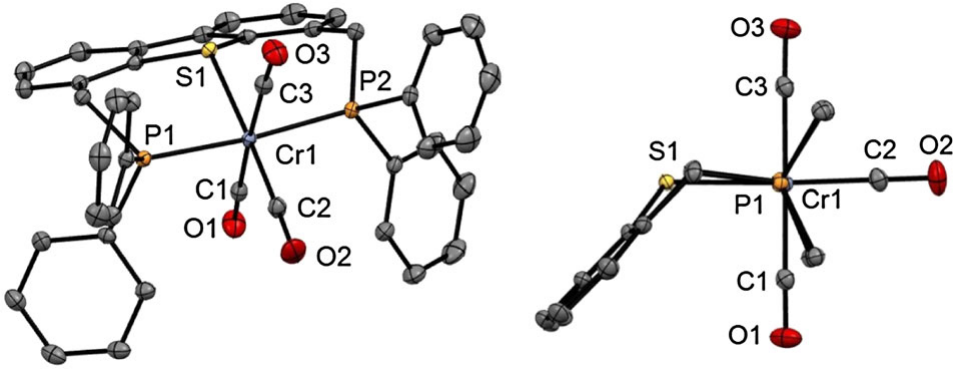
(*Left*) Structural view of [Cr(κ^3^ *P,S,P*-PSP^Ph^)(CO)_3_] (**4**) showing 50 % thermal ellipsoids (hydrogen atoms and solvent omitted for clarity). Selected bond lengths (Å) and angles (°): Cr1–C1 1.891(1), Cr1–C2 1.834(1), Cr1–C3 1.888(1), Cr1–P1 2.3226(4), Cr1–P2 2.3283(4), Cr1–S1 2.3414(4), P1–Cr1–P2 174.82(1), S1–Cr1–C2 176.52(5), C1–Cr1–C3 176.79(6). (*Right*) Structural view of the inner coordination sphere of **4**

Complex **4** was fully characterized by a combination of ^1^H and ^31^P{^1^H} NMR spectroscopy, IR spectroscopy, ESI MS, and elemental analysis. Due to the poor solubility of this complex a useful ^13^C{^1^H} NMR spectrum could not be obtained. The ^31^P{^1^H} NMR spectra exhibit singlet resonances at 79.3 ppm (cf. −17.3 ppm in the free ligand **3**). The IR spectrum exhibits two strong bands at 1820 and 1850 cm^−1^ assignable to the symmetric and the two superimposed strong asymmetric ν_CO_ stretching modes. In addition the molecular structure of **4** was determined by X-ray crystallography. A structural view is depicted in Fig. 1 with selected bond distances and angles given in the caption. The coordination geometry around the chromium center corresponds to a slightly distorted octahedron with the PSP ligand coordinated in the typical meridional κ^3^*P,S,P* bonding mode. The P1-Cr1-P2, S1-Cr1-C2, and C1-Cr1-C3 angles deviate from 180° being 174.82(1)°, 176.52(5)°, and 176.79(6)°, respectively. As expected, the Cr-C distances of the CO ligands *trans* to one another are slightly longer (1.891(1) and 1.888(1) Å) than the one *trans* to the thiophene moiety (1.834(1) Å) due the strong *trans* influence of the CO ligand.

Since ESI–MS enables not only the detection and the study of reaction substrates and products but also short-lived reaction intermediates and decomposition products as they are present in solution, complex **4** was investigated by means of this technique. A methanolic solution of **4** in the presence of NaCl was subjected to ESI–MS analysis in the positive ion mode.

**Fig. 2:**
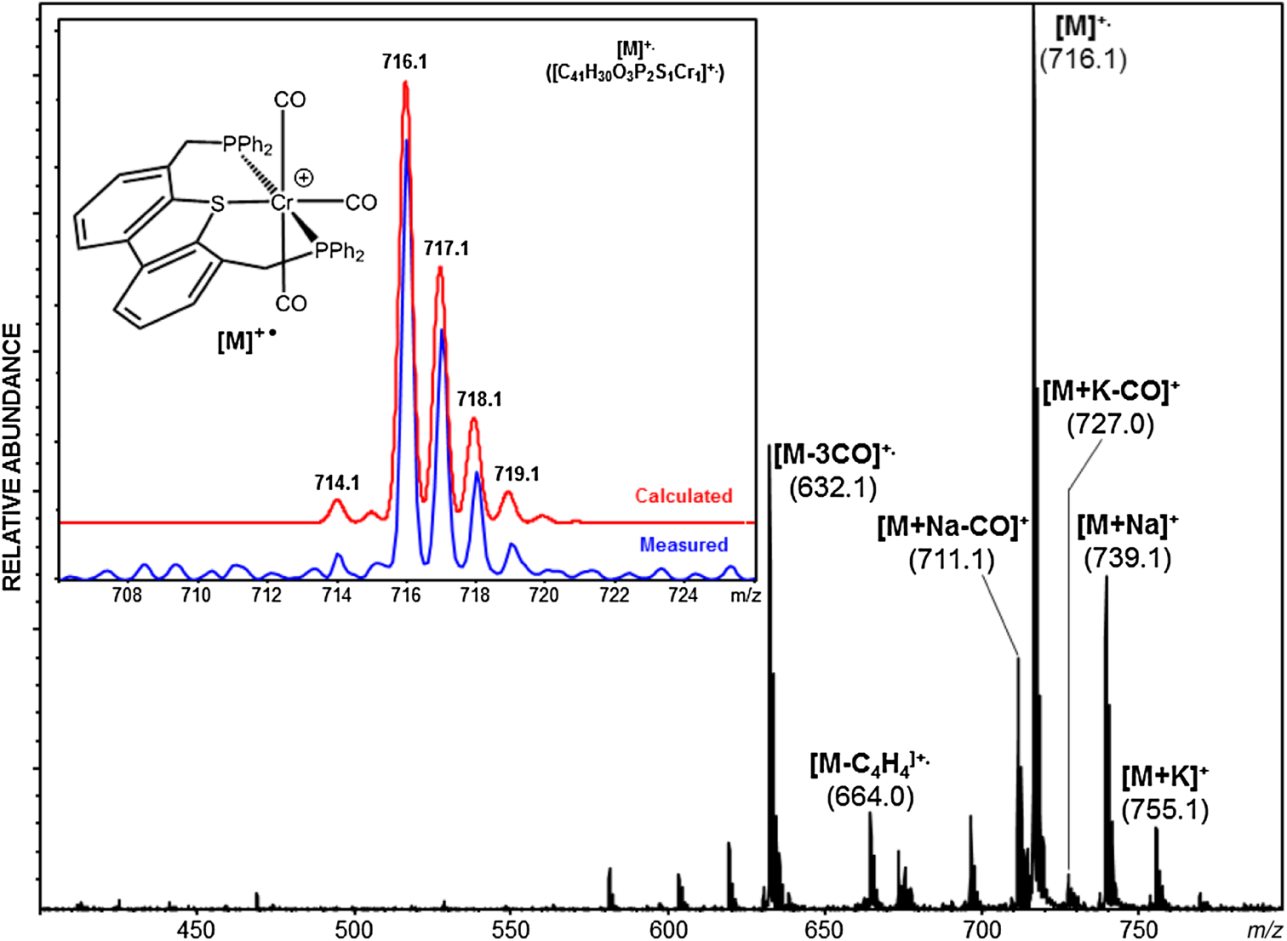
Positive-ion ESI full scan mass spectrum of [Cr(κ^3^ *P,S,P*-PSP^Ph^)(CO)_3_] (**4**) in CH_3_OH. Inset shows the calculated and measured isotopic pattern of the radical cation [M]^+·^. All mass calculations and mass assignments are based on the most abundant chromium isotope ^52^Cr

These measurements revealed that complex **4** remains intact and also [M + Na]^+^ and [M + K]^+^ ions were observed at *m/z* = 739.0 and 754.9, respectively. The most abundant ion related to the molecular ion is the radical cation [Cr(κ^3^*P,S,P*-PSP^Ph^)(CO)_3_]^+^ [M]^+·^ at *m/z* = 716.0 as a result of oxidation of the metal center or the thiophene moiety. In addition, fragments of [M + Na-CO]^+^ and [M + K-CO]^+^ were detected at *m/z* = 711.0 and 727.0, respectively, due to dissociation of one CO ligand. The full scan ESI–MS spectrum of **4** in methanol is depicted in Fig. 2. In the inset, the isotopic pattern of the radical cation [M]^+·^ is compared with the theoretical pattern, which turned out to correlate quite well.

In sum, the first benzothiophene PSP pincer ligand was prepared. The Cr(0) tricarbonyl complex [Cr(κ^3^*P,S,P*-PSP^Ph^)(CO)_3_] was synthesized via a solvothermal reaction and was fully characterized by a combination of ^1^H, ^13^C{^1^H}, and ^31^P{^1^H} NMR spectroscopy, IR spectroscopy, ESI MS, and elemental analysis.

## Experimental

All manipulations were performed under an inert atmosphere of argon by using Schlenk techniques or in a MBraun inert-gas glovebox. 4,6-Bis(hydroxymethyl)dibenzothiophene (**1**) was prepared according to the literature [11, 12]. The solvents were purified according to standard procedures [13]. The deuterated solvents were purchased from Aldrich and dried over 4 Å molecular sieves. ^1^H, ^13^C{^1^H}, and ^31^P{^1^H} NMR spectra were recorded on a Bruker AVANCE-250 spectrometer operating at 250.13, 62.86, and 101.26 MHz. ^1^H and ^13^C{^1^H} NMR spectra were referenced internally to residual protio-solvent, and solvent resonances, respectively, and are reported relative to tetramethylsilane (*δ* = 0 ppm). ^31^P{^1^H} NMR spectra were referenced externally to H_3_PO_4_ (85 %) (*δ* = 0 ppm). As reaction vessel 20 cm^3^ microwave vials from Biotage or VWR with an aluminium septum cap were used.

All mass spectrometric measurements were performed on an Esquire 3000^*plus*^ 3D-quadrupole ion trap mass spectrometer (Bruker Daltonics, Bremen, Germany) in positive-ion mode by means of electrospray ionization (ESI). Mass calibration was done with a commercial mixture of perfluorinated trialkyl-triazines (ES Tuning Mix, Agilent Technologies, Santa Clara, CA, USA). All analytes were dissolved in CH_3_OH “hypergrade for LC–MS Lichrosolv” quality (Merck, Darmstadt, Germany) to form a concentration of roughly 1 mg/cm^3^ and doped with sodium chloride to promote the corresponding [M + Na]^+^ ion formation. Direct infusion experiments were carried out using a Cole Parmer model 74900 syringe pump (Cole Parmer Instruments, Vernon Hills, IL, USA) at a flow rate of 2 mm^3^/min. Full scan and MS/MS (low energy CID)-scans were measured in the range *m/z* = 100–1100 with the target mass set to *m/z* = 1000. Further experimental conditions include: drying gas temperature: 150 °C; capillary voltage: −4 kV; skimmer voltage: 40 V; octapole and lens voltages: according to the target mass set. All mass calculations are based on the most abundant metal isotope ^52^Cr isotope. Mass spectra were averaged during data acquisition time of 1–2 min and one analytical scan consisted of five successive micro scans resulting in 50 and 100 analytical scans, respectively, for the final full scan mass spectrum.

### *4,6*-*Bis(bromomethyl)dibenzothiophene* (**2**, C_14_H_10_Br_2_S)

A solution of 5.0 g 4,6-bis(hydroxymethyl)dibenzothiophene (**1**, 20.5 mmol) in 200 cm^3^ chloroform was cooled to 0 °C and 4.8 cm^3^ PBr_3_ (51.2 mmol) was added in a dropwise fashion. The solution was allowed to reach room temperature and was stirred for 2 h. After that the reaction was quenched with ice water and the product was extracted with chloroform. The organic layer was washed with water and then dried over Na_2_SO_4_. Removals of the solvent under reduced pressure afforded **2** as a light yellow solid. Yield: 5.0 g (67 %); ^1^H NMR (DMSO-*d*_*6*_): *δ* = 8.38 (d, *J*_HH_ = 7.8 Hz, 2H, Ar), 7.69 (t, *J*_HH_ = 7.3 Hz, 2H, Ar), 7.55 (t, *J*_HH_ = 7.4 Hz, 2H, Ar), 5.02 (s, 4H, CH_2_) ppm; ^13^C{^1^H} NMR (DMSO-*d*_*6*_): *δ* = 138.2 (C_Thio_), 136.0 (C_Thio_), 132.0 (Ph), 128.4 (Ph), 125.5 (Ph), 122.7 (Ph), 33.1 (CH_2_) ppm.

### *4,6*-*Bis(diphenylphosphinomethyl)dibenzothiophene (PSP*^*Ph*^*)* (**3**, C_38_H_30_P_2_S)

Diphenylphosphine (0.94 cm^3^, 5.4 mmol) was dissolved in 15 cm^3^ THF and cooled to −78 °C. Then 2.2 cm^3^*n*-BuLi (2.5 M in hexanes, 5.4 mmol) was slowly added and the mixture was allowed to reach room temperature and was stirred for 1 h. The solution was again cooled to −78 °C and 0.96 g 4,6-bis(bromomethyl)dibenzothiophene (**2**, 2.6 mmol) suspended in 10 cm^3^ dry THF was added and stirred for 3 h while allowing to reach room temperature. The solvent was then removed under vacuum, the residue dissolved in toluene, insoluble materials were removed by filtration over Celite. Evaporating to dryness afforded **3** as a white solid. Yield: 1.37 g (91 %); ^1^H NMR (250 MHz, DMSO-*d*_*6*_): *δ* = 8.12 (d, *J* = 7.83 Hz, 2H, ArH), 7.52–7.45 (m, 10H, ArH), 7.37–7.35 (m, 10H, ArH), 7.29 (t, *J* = 7.3 Hz, 2H, ArH), 6.06 (d, *J* = 7.4 Hz, 2H, ArH), 3.77 (bs, CH_2_, 4H) ppm; ^13^C{^1^H} NMR (DMSO-*d*_*6*_): *δ* = 138.9 (vt, *J* = 3.8 Hz, C_Thio_), 138.3 (d, *J* = 15.6 Hz, Ph), 136.3 (d, *J* = 1.4 Hz, C_Thio_), 133.2 (d, *J* = 19.0 Hz, PhH), 132.4 (vd, *J* = 9.4 Hz, C_Thio_), 129.5 (PhH), 129.0 (d, *J* = 6.7 Hz, PhH), 127.9 (d, *J* = 7.8 Hz, CH_Thio_), 125.3 (CH_Thio_), 120.3 (CH_Thio_), 35.04 (d, *J*_CP_ = 17.1 Hz, CH_2_) ppm; ^31^P{^1^H} NMR (CDCl_3_): *δ* = −17.6 ppm; HRMS (ESI): *m/z* = 581.1625 ([M + H]^+^), C_38_H_31_P_2_S requires 581.1622.

### *[(4,6*-*Bis(diphenylphosphinomethyl)dibenzothiophene)(tricarbonyl)chromium(0)][Cr(κ*^*3*^*P,S,P*-*PSP*^*Ph*^*)(CO)*_*3*_*]* (**4**, C_41_H_30_CrO_3_P_2_S)

4,6-Bis(diphenylphosphinomethyl)dibenzothiophene (PSP^Ph^) (**3**, 137 mg, 0.24 mmol) and 44 mg [Cr(CO)_6_] (0.21 mmol) were suspended in 7 cm^3^ CH_3_CN in a sealed tube and heated for 6 h at 140 °C. From this solution complex **4** crystalized upon cooling to room temperature as small orange needles. Yield: 133 mg (89 %); ^1^H NMR (DMSO-*d*_*6*_): *δ* = 8.18–8.16 (m, 2H, Ar), 7.71 (br, 6H, Ar), 7.52–7.48 (m, 6H, Ar), 7.41–7.30 (m, 13H, Ar), 3.78 (bs, CH_2_, 4H) ppm; ^31^P{^1^H} NMR (DMSO-*d*_*6*_): *δ* = 79.3 ppm; IR (ATR): $\bar{V}$ = 1820 (ν_C=O_), 1850 (ν_C=O_) cm^−1^.

### X-ray structure determination

Crystals of **4** of good optical quality were pre-selected, embedded in perfluorinated polyether and mounted on MiTeGen MicroLoops (CCDC 1446055). X-ray diffraction data were measured using *ω*- and *φ*-scans at *T* = 100 K on a Bruker APEX-II diffractometer with Mo-*K*α radiation. The collection strategy for the measurement was optimized with the APEX-2 software [14] to result in a data set of the complete reciprocal sphere up to high angles and with high completeness. After integration of the data with the program SAINT [14], an absorption correction based on the semi-empirical “multi-scan” approach was performed with the SADABS program [14]. The crystal structure was solved by direct methods and was refined using the SHELXTL program package [15]. All H atoms were placed geometrically and refined in the riding model approximation, with C–H = 0.95 Å for aromatic H atoms (C–H = 0.99 Å for methylene H atoms) and with *U*_iso_(H) = 1.2 *U*_eq_(C). The crystal contained acetonitrile solvent molecules disordered around an inversion centre. In the final model, the occupancy of each atom of the acetonitrile solvent molecule was constrained to 0.5. All non-hydrogen atoms were refined anisotropically. The methyl H atoms of the solvent molecule were not modelled but are included in the formula of the compound. Molecular graphics were generated with the program MERCURY [16].

## Electronic supplementary material

Below is the link to the electronic supplementary material.

Supplementary material 1 (CIF 21 kb)
